# Characterization of larval growth in *C. elegans* cuticle mutants

**DOI:** 10.17912/micropub.biology.000662

**Published:** 2022-11-04

**Authors:** Joy Nyaanga, Sasha Shirman, Niall M. Mangan, Erik C. Andersen

**Affiliations:** 1 Department of Molecular Biosciences, Northwestern University, Evanston, IL 60208, USA; 2 Interdisciplinary Biological Sciences Program, Northwestern University, Evanston, IL 60208, USA; 3 Department of Engineering Sciences and Applied Mathematics, Northwestern University, Evanston, IL 60208, USA

## Abstract

In *Caenorhabditis elegans,* many genes involved in the formation of the cuticle are also known to influence body size and shape. We assessed post-embryonic growth of both long and short *C. elegans* body size mutants from the L1 to L4 stage. We found similar developmental trajectories of N2 and *lon-3* animals. By contrast, we observed overall decreases in body length and increases in body width of tested *dpy* mutants compared to N2, consistent with the Dpy phenotype. We further show that the dynamics of animal shape in the mutant strains are consistent with a previously proposed “Stretcher” growth model.

**Figure 1. Quantitative assessment of larval growth:**
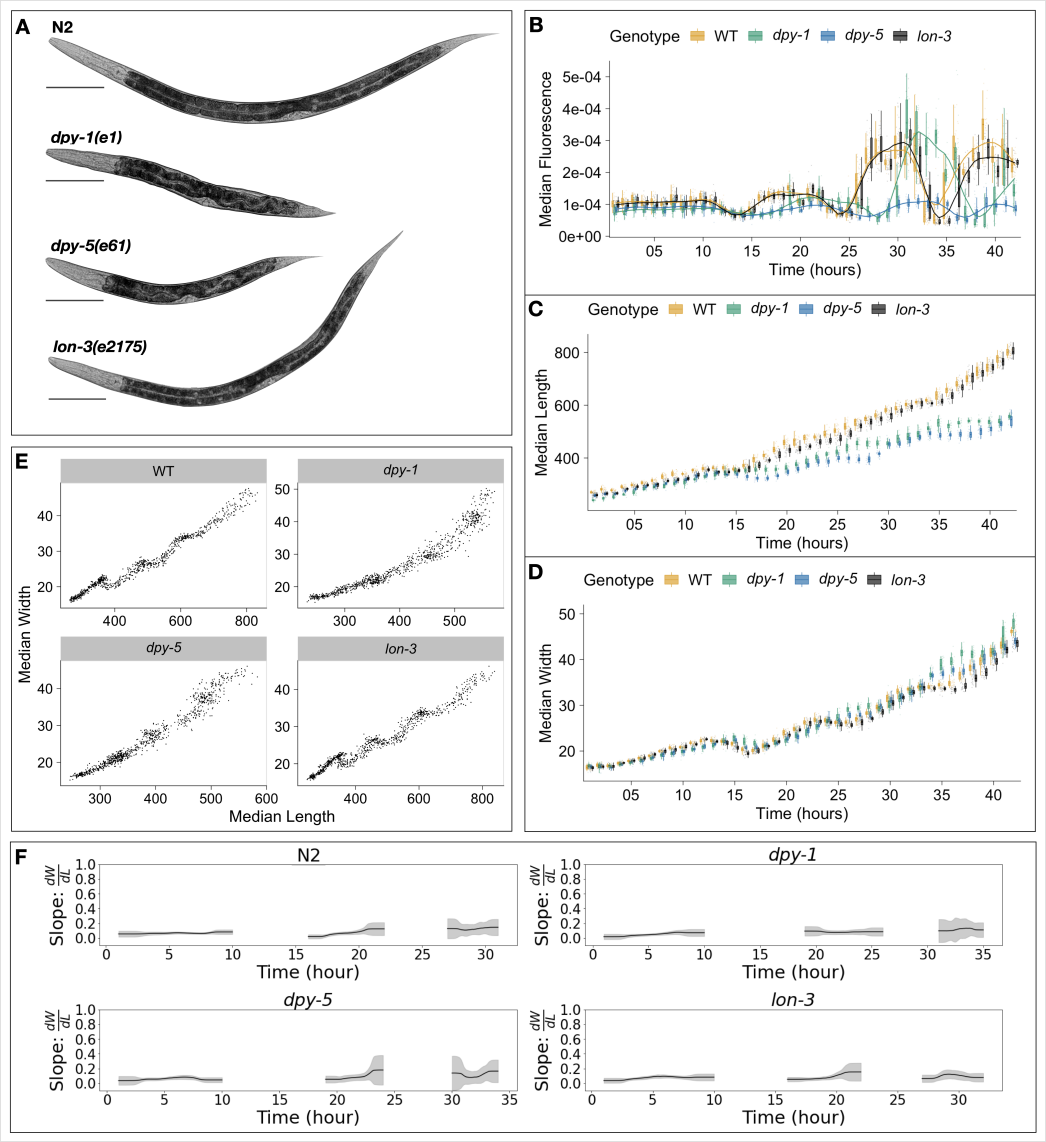
**(A)** Representative images of strains used in this study taken at the L4 stage. **(B)** Tukey boxplots for median animal fluorescence normalized by area, **(C)** median animal length (µM), and **(D)** median animal width (µM). The horizontal line in the middle of the box is the median, and the box denotes the 25th to 75th quantiles of the data. The vertical line represents the 1.5 interquartile range. Each point corresponds to the median value of a population of animals in each well. **(E)** Median length (x-axis) plotted against median length (y-axis). **(F)** The ratio of the change in width to length over time, calculated from the local slope of the population data, is shown as a solid line. The standard deviation, represented as the shaded region, captures population variation.

## Description

Body size largely influences a variety of organismal traits, including: growth, reproduction, metabolism, and lifespan. For this reason, the factors that control organism size, particularly during development, have been explored in many systems. *Caenorhabditis elegans* , a free-living nematode, presents a versatile genetic model system to study how the processes of growth and development are regulated. *C. elegans* matures to an adult after multiple molting events during which time animals synthesize a new exoskeleton (cuticle) and expel their old one. This cuticle is a complex, multi-layered structure primarily composed of collagens. As animals progress through their life-cycle, the structure and thickness of the cuticle changes but its role in the maintenance of body morphology and integrity remains. To date, 21 cuticle collagen mutants have been identified that cause a range of body morphology defects (Page and Johnstone 2007). Some of these mutants exhibit a disproportionate reduction in body size compared to the wild type, and others are noticeably larger than the wild type (Cho et al. 2021), clearly demonstrating the importance of the physical structure of the cuticle on growth. Analyzing the characteristics of size during development in various *C. elegans* body shape mutants is central to understanding the role these genes have on body growth.

We performed a high-resolution longitudinal study of growth in a selection of *C. elegans* cuticle mutants. We chose mutants that were both reportedly shorter ( *dpy-1(e1), dpy-5(e61)* ) and longer ( *lon-3(e2175)* ) than the wild type (Cho et al. 2021) ( **Figure 1A** ). We then collected high-precision measurements of animal fluorescence ( **Figure 1B** ), length ( **Figure 1C** ), and width ( **Figure 1D** ) from the L1 stage into the L4 stage. As we previously demonstrated (Nyaanga et al. 2022), by exposing animals to ingestible fluorescent beads, we can use oscillations in fluorescence as a proxy for feeding behavior. To characterize larval progression, periods of decreased feeding are associated with molt events. Doing so, we noticed that *lon-3(e2175* ) animals follow similar developmental trajectories to the N2 wild-type strain. By contrast, we detected a delay in the molt timing of both *dpy* mutant strains, with *dpy-1(e1* ) undergoing each larval transition later than all other tested strains. We also observed a marked decrease in animal length and increase in animal width noticeable after the L1 stage in both *dpy* strains, consistent with their characteristic Dpy phenotype. Interestingly, we noted little size divergence between *lon-3(e2175)* and N2 animals during our time course.

Measurements of both animal length and width allowed us to assess changes in body shape in addition to size. Motivated by changes in the body aspect ratio of animals that we observed at larval-stage transitions, we previously modeled a physical mechanism by which constraints on cuticle stretch could cause changes in *C. elegans* body shape, and found that model-predicted shape changes were consistent with data from the N2 wild-type strain (Nyaanga et al. 2022). Given this result, we proposed a “Stretcher” model for growth wherein *C. elegans* sense changes in cuticle elasticity, in tandem with other regulatory mechanisms, to control growth rate and determine developmental transitions. Given the structural impacts caused by the *dpy-1(e1), dpy-5(e61)* , and *lon-3(e2175)* mutations, we sought to determine whether the shape dynamics predicted by the Stretcher model would be consistent with the mutant data. By analyzing the relationship between measured animal length and width over time, we were able to detect the linear stretch, nonlinear stretch, and relaxation regimes predicted by the Stretcher model ( **Figure 1F** ) and observed in our previous work (Nyaanga et al. 2022). If the cuticle responds to internal pressure from growth linearly, then the ratio of change in width and length (W/L) is expected to be constant. If the cuticle stretches beyond some threshold, it exhibits nonlinear response and the ratio will change as the cuticle becomes more stiff in either length or width direction. The linear regime indicated by constant slope at the start of the larval stage and nonlinear stretch regimes indicated by an increase in slope at the end of the larval stage were most apparent in the L2 stage of the N2 strain, *lon-3(e2175)* , and *dpy-5(e61)* . We did not observe as significant an increase in slope in *dpy-1(e1)* , possibly due to changes in cuticle elasticity for this mutant, or in the other larval stages, possibly due to challenges in measuring smaller L1 animals and population desynchronization in the L3 stage as animals age. Following the non-linear stretch regime, we expect to observe a post-molt relaxation as the old stretch-restricted cuticle is shed and the animal changes shape to fill the new cuticle. Notably, although we observed a post-molt relaxation period in all strains, we found differences in the *dpy* mutants when compared to the N2 strain and the *lon-3(e2175)* mutant ( **Extended Data** ). In *dpy-1(e1)* animals we see post-molt increase in length accompanied by a corresponding decrease in width, consistent with dynamics observed in the N2 strain and *lon-3(e2175)* animals. However, we noted a marked decrease in the magnitude of relaxation. By contrast, in *dpy-5(e61)* animals, we noticed a change in the direction of relaxation with animals appearing to exhibit post-molt relaxation in width as opposed to length. This difference in relaxation could be a result of changes in cuticle stiffness caused by the *dpy-5(e61)* mutation, as previous work has revealed that *dpy-5(e61)* animals are less stiff than N2 animals (Park et al. 2007).

This study investigated the influence of cuticle mutations on *C. elegans* larval growth. We found that the *dpy-1(e1)* or *dpy-5(e61)* mutations strongly impacted organism size and developmental speed. Although it is unclear from our work how much each mutant could interfere with signaling processes associated with molt timing, it is evident that cuticle properties strongly influence growth dynamics at larval-stage transitions.

## Methods

**Worm culture**

The laboratory strain N2 was obtained from the *C. elegans* Natural Diversity Resource (Cook et al. 2017). All other strains were provided by the CGC, which is funded by NIH Office of Research Infrastructure Programs (P40 OD010440). Animals were cultured at 20°C on 6 cm plates of modified nematode growth media (NGMA), which contained 1% agar and 0.7% agarose seeded with *E. coli* OP50 bacteria (Andersen et al. 2014).

**High-throughput growth assay**

Measurements of body size and fluorescence were measured as previously described (Nyaanga et al. 2022). Briefly, strains were propagated for three generations, bleach-synchronized, and titered at a concentration of 1 embryo per µL into 250 mL flasks for a total of 250,000 embryos. The following day, arrested L1s were fed HB101 food at a final concentration of OD20 in a final flask volume of 100 mL K medium and HB101 food. Animals were grown with constant shaking at 20°C. Flasks were sampled each hour beginning one hour after feeding and continuing for 42 consecutive hours. At each hour, 800 µL was removed from each flask and incubated with fluorescent polychromatic beads (Polysciences, 19507-5) for 10 minutes with shaking. Following the bead incubation, animals were transferred to a 96-well microtiter plate, treated with sodium azide, imaged with an ImageXpress Nano (Molecular Devices, SanJose, CA), and scored using a large-particle flow cytometer (COPAS BIOSORT, Union Biometrica, Holliston MA). COPAS BIOSORT was used to collect measurements of animal length (TOF), optical extinction (EXT), and red fluorescence for every animal in each well.

**Data processing**

COPAS BIOSORT data were processed as previously described (Nyaanga et al. 2022). To remove non-animal objects such as bacterial clumps, shed cuticles, and next-generation larval animals from the time-course data. Data for each well were summarized, generating median well measurements. TOF and norm.EXT data were then converted to microns. This conversion was performed by first collecting manual measurements of animal size from images using the free Java image-processing program ImageJ (Schneider et al. 2012). Briefly, length was measured from head to tail, and width was measured at the widest point of the animal. Five animals were measured per well across thirty total wells for each hour. Pixels were converted to µm using a conversion factor of 3.2937 pixels/µm. Only the unit-corrected data were used for further analysis.

“Stretcher” model analysis of shape dynamics was performed as previously described (Nyaanga et al. 2022). Briefly, length and width data were extracted from the full population data by selecting time periods that were in active growth and omitting time periods where post-molt relaxation could be observed. The hours defining these growth periods for the L1 stage were, 1-10. For the L2 stage the hours were: N2 and *lon-3* : 16-22, *dpy-1* : 19-26, and *dpy-5* : 19-24. Lastly, the L3 hours were: N2: 27-31, *lon-3* : 27:32, *dpy-1* : 31-35, and *dpy-5* : 30-34. The L4 stage was excluded because of high variability in the population. We next applied a local kernel regression to smooth the population measurements and calculated mean and standard deviation using bootstrapping. The ratio of the change in width to length over time, calculated from the local slope of the population data, was calculated to determine cuticle properties across larval stages. All files and code for analysis and generation of figures and tables are archived on GitHub ( https://github.com/AndersenLab/cuticle-mutants-micropub ).

## Reagents

**Table:** 

Strain	Genotype	Available From
N2	*Caenorhabditis elegans*	CeNDR
CB1	*dpy-1(e1)*	CGC
CB61	*dpy-5(e61)*	CGC
CB4123	*lon-3(e2175)*	CGC

## Extended Data

Description: Median length (x-axis) is plotted against median length (y-axis) to visualize changes in body aspect ratio over time. Each point corresponds to the median length or median width of animals at each experimental hour. Post-molt relaxation is observed in WT, lon-3(e2175), and dpy-1(e1) as a slight decrease in median animal width. Post-molt relaxation is observed in dpy-5(e61) as a slight decrease in animal length. . Resource Type: Audiovisual. DOI: 10.22002/d60qd-yy629
